# Supplementary material for: EnzML: multi-label prediction of enzyme classes using InterPro signatures
Source: BMC Bioinformatics. 2012 Apr 25;13:61. doi: 10.1186/1471-2105-13-61 (PMC3483700; doi:10.1186/1471-2105-13-61)
Supplement: Addtional file 5 — The Java code to format the data files, evaluate and predict. The file enzml_java_code.tar.gz contains the Java code used to format database data to ARFF and XML formats, to execute cross and train-test (jackknife) evaluations and to record evaluation results to database. More information is included in the readme.txt file and the Javadoc files. The code can be used with a MySQL database. To use a different database software, other JDBC drivers might be required. [file 1471-2105-13-61-S5.gz › java_code/enzml2011/doc/uk/ac/ed/inf/taweka/mulan/attributesfilter/AttributesFilter.html]

AttributesFilter


---


|  |  |  |
| --- | --- | --- |
| **Overview** \| **Package** \| **Class** \| **Use** \| **Tree** \| **Deprecated** \| **Index** \| **Help** | |  |
| PREV CLASS   **NEXT CLASS** | **FRAMES**    **NO FRAMES**     **All Classes** |  |
| SUMMARY: NESTED \| FIELD \| CONSTR \| METHOD | DETAIL: FIELD \| CONSTR \| METHOD |  |


---


## uk.ac.ed.inf.taweka.mulan.attributesfilter Class AttributesFilter

```
java.lang.Object
  uk.ac.ed.inf.taweka.Initialised
      uk.ac.ed.inf.taweka.mulan.attributesfilter.AttributesFilter
```

**All Implemented Interfaces:**: Initialisable

---

``` public class AttributesFilter extends Initialised ```

This class filters the attributes of a dataset (the test set) to make it
compatible with another dataset (the train set). The result is a new dataset
that can be used as test set for the specified train set.
Main operations: 1. Attributes: only attributes present in the train set can
appear in the test set (& all of them must be present) 2. Class attributes:
for the test set are irrelevant, but are usually set to '?'. 3. Mulan XML
file (class labels): the same XML file of the training set 4. ARFF File: the
new test set arff file name should contain 'train name' + 'test name' +
'.test' 5. ARFF record: if stored in the database, should contain trace
(id/props file path) of the original training set and the other set used to
generate the new test set.

**Version:**
:   3 Sep 2010

**Author:**
:   Luna De Ferrari luna.deferrari-at-ed.ac.uk

---

**Field Summary**

**Fields inherited from class uk.ac.ed.inf.taweka.Initialised**

`m_initialisationIsCorrect`


**Constructor Summary**

`AttributesFilter(int trainArffId,
int oldTestArffId,
java.lang.String arffDatabasePropsPath,
java.lang.String resultsPropsFileName)`


| **Method Summary** | |
| --- | --- |
| `int` | `createNewTestArff()` |
| `AttributesFilteredArff` | `getNewTestArff()` |
| `MulanArff` | `getOldTestArff()` |
| `int` | `getOldTestSetArffId()` |
| `java.lang.String` | `getResultsProperties()` |
| `MulanArff` | `getTrainArff()` |
| `int` | `getTrainSetArffId()` |
| `java.lang.String` | `getTrainXmlPath()` |
| `java.lang.String` | `getXmlPathById(int arffId)` |
| `static void` | `main(java.lang.String[] args)` |
| `void` | `writeNewTestArff()` |

**Methods inherited from class uk.ac.ed.inf.taweka.Initialised**

`initialisationIsCorrect, setInitialisationIsCorrect`

**Methods inherited from class java.lang.Object**

`equals, getClass, hashCode, notify, notifyAll, toString, wait, wait, wait`

**Constructor Detail**

### AttributesFilter

```
public AttributesFilter(int trainArffId,
                        int oldTestArffId,
                        java.lang.String arffDatabasePropsPath,
                        java.lang.String resultsPropsFileName)
```


**Method Detail**

### createNewTestArff

```
public int createNewTestArff()
```

---


### getNewTestArff

```
public AttributesFilteredArff getNewTestArff()
```

---


### getOldTestArff

```
public MulanArff getOldTestArff()
```

---


### getOldTestSetArffId

```
public int getOldTestSetArffId()
```

---


### getResultsProperties

```
public java.lang.String getResultsProperties()
```

---


### getTrainArff

```
public MulanArff getTrainArff()
```

---


### getTrainSetArffId

```
public int getTrainSetArffId()
```

---


### getTrainXmlPath

```
public java.lang.String getTrainXmlPath()
```

---


### getXmlPathById

```
public java.lang.String getXmlPathById(int arffId)
```

---


### writeNewTestArff

```
public void writeNewTestArff()
```

---


### main

```
public static void main(java.lang.String[] args)
```


---


|  |  |  |
| --- | --- | --- |
| **Overview** \| **Package** \| **Class** \| **Use** \| **Tree** \| **Deprecated** \| **Index** \| **Help** | |  |
| PREV CLASS   **NEXT CLASS** | **FRAMES**    **NO FRAMES**     **All Classes** |  |
| SUMMARY: NESTED \| FIELD \| CONSTR \| METHOD | DETAIL: FIELD \| CONSTR \| METHOD |  |


---
